# Methoxyflurane Instead of Morphine in Acute Coronary Syndrome Patients: Results of the Randomized Controlled METHANE-SIRIO 4 Study

**DOI:** 10.1055/a-2698-3958

**Published:** 2025-09-18

**Authors:** Piotr Niezgoda, Przemysław Podhajski, Michał Kasprzak, Malwina Barańska, Oscar Rakotoarison, Bożena Karolko, Łukasz Pietrzykowski, Piotr Michalski, Agata Kosobucka, Adam Sikora, Joanna Chałupka, Michał Marszałł, Wiktor Kuliczkowski, Aldona Kubica, Jacek Kubica

**Affiliations:** 1 Department of Cardiology and Internal Medicine, Collegium Medicum, Nicolaus Copernicus University, Bydgoszcz, Poland; 2 Institute for Heart Diseases, Wroclaw Medical University, Wroclaw, Poland; 3 Department of Health Promotion, Collegium Medicum, Nicolaus Copernicus University, Bydgoszcz, Poland; 4 The Department of Medicinal Chemistry, Collegium Medicum, Nicolaus Copernicus University, Bydgoszcz, Poland; 5 Department of Pharmaceutical Technology, Faculty of Pharmacy, Medical Biotechnology and Laboratory Medicine, Pomeranian Medical University, Szczecin, Poland

**Keywords:** acute coronary syndrome, unstable angina, methoxyflurane, morphine, platelet reactivity

## Abstract

**Aim:**

Morphine is known to negatively influence the pharmacokinetics (PK) and the antiplatelet effect of oral P2Y12 receptor inhibitors administered due to an acute coronary syndrome. Worldwide studies of the potential approaches to overcome the undesired morphine effect have been only partially successful. The aim of the METHANE-SIRIO 4 study was to evaluate the PK and pharmacodynamics of ticagrelor and its active metabolite in unstable angina (UA) patients treated with morphine or methoxyflurane.

**Methods and Results:**

Consecutive patients diagnosed with UA, randomized into the study arms, underwent the assessment of platelet reactivity using the Multiplate analyzer at nine predefined study time points. Serum concentrations of ticagrelor and its active metabolite, AR-C124910XX, were measured for each study participant. Median platelet reactivity was significantly lower in patients who received ticagrelor and methoxyflurane versus ticagrelor alone at 30 minutes postticagrelor loading dose. A trend toward lower reactivity was observed at 45 and 240 minutes. Significant differences in median serum concentrations of ticagrelor and its active metabolite were most pronounced between patients treated with ticagrelor and methoxyflurane versus ticagrelor and morphine.

**Conclusion:**

Co-administration of methoxyflurane in acute coronary syndrome patients allows for the avoidance of negative effects of morphine and has the additional effect of inhibiting platelet reactivity. Further randomized studies would be recommended to support these findings.

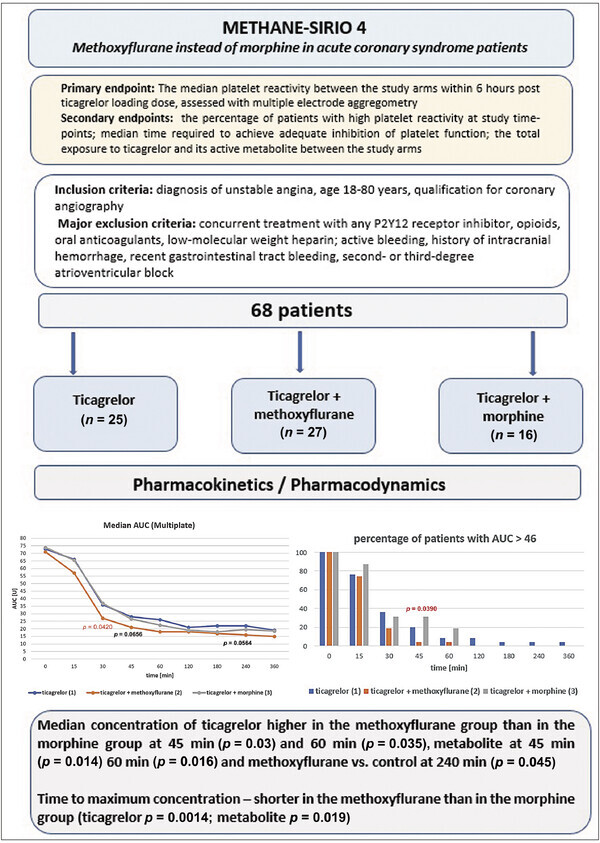

## Introduction

Platelet adhesion, activation, and aggregation are among the main pathophysiological processes of coronary thrombosis in the course of acute coronary syndromes and thrombotic complications in patients treated with percutaneous coronary intervention (PCI). 1 Oral antiplatelet drugs, including aspirin and P2Y12 receptor inhibitors, together with heparin and percutaneous coronary angioplasty, are currently the standard of care for acute coronary syndromes. According to the guidelines of the European Society of Cardiology, among P2Y12 receptor inhibitors, ticagrelor and prasugrel are preferred. 2 To relieve angina symptoms in patients with acute coronary syndrome, morphine is administered. However, based on the previously published data, patients receiving morphine analgesia due to ischemic pain are known to have not only a weaker, but also a delayed response to antiplatelet agents such as ticagrelor, clopidogrel, prasugrel, but not aspirin. 3 4 5 Notably, several other medical conditions, including cardiogenic shock or gastrointestinal motility disorders, may worsen the pharmacokinetics (PK) or pharmacodynamics (PD) of P2Y12 receptor inhibitors, even resulting in persistent hyperactivation of platelets despite antiaggregatory pharmacotherapy. 6 7 Nevertheless, the latest ESC guidelines recommend intravenous administration of opioid analgesics for acute pain management in ACS patients, as a class IIa recommendation owing to proven inferiority of alternative approaches, including the administration of fentanyl, acetaminophen, or nitrous oxide. 2 8 9 While morphine is known to negatively impact the gastrointestinal motility, inhibit gastric emptying, reduce gut secretion, and reduce peristalsis of the intestines, the need to overcome the so-called “morphine effect” emerged. 10 Apart from substitution of morphine as an analgesic agent, several other methods, including sublingual administration of crushed ticagrelor, administration of crushed clopidogrel via a nasogastric tube, co-administration of intravenous metoclopramide or oral naloxone, have been examined to date. While the administration of crushed P2Y12 receptor inhibitors was found to be beneficial in terms of optimization of their PK/PD profile, the utility of the remaining methods is rather limited. 11 12 13 14 Therefore, there is an urgent need to look for a new way to solve this old problem.

Methoxyflurane is a nonopioid inhaled anesthetic, historically used in the induction of general anesthesia. With its registration in severe trauma, it is used in emergency medicine. In the face of its mechanism of action, it has been put forward that co-administration of methoxyflurane in ACS patients should not exert negative gastrointestinal effects, as observed in individuals treated with morphine. Moreover, methoxyflurane is characterized by a safe cardiovascular profile, without the increased risk of nephrotoxicity or hepatotoxicity. Only mild and generally easily reversible adverse effects, comprising dizziness, headache, or somnolence, can be expected. 15

The aim of this study was to evaluate the PK/PD profile of ticagrelor and its active metabolite, AR-C124910XX, in patients presenting with unstable angina (UA) who received methoxyflurane/morphine or no analgesia. Simultaneously, a pilot study testing the analgesic efficacy of methoxyflurane in patients with a diagnosis of an acute myocardial infarction was launched (ANEMON-SIRIO 3 study). 16

## Methods

The METHANE-SIRIO 4 study was designed as a phase 4, randomized, open-label study aimed at evaluating the PK/PD profile of ticagrelor in patients diagnosed with UA. The study was conducted in the Department of Cardiology of the Nicolaus Copernicus University and the Department of Cardiology of Wrocław Medical University. The study protocol was approved by the Ethics Committee of the Nicolaus Copernicus University with the approval number KB 37/2020. All study-related procedures were performed in full accordance with the regulations of The Declaration of Helsinki and Good Clinical Practice. The study was registered on clinicaltrials.gov (NCT 04442919).

All patients diagnosed with UA aged 18 to 80 years, admitted to the study sites, who were qualified for coronary angiography, were screened for eligibility. The enrolled participants were randomized in a 1:1:1 ratio using the Random Allocation Software 2.0 into the study arms as follows: (1) patients who received a loading dose (LD) of ticagrelor (180 mg)—control group, (2) patients who received ticagrelor LD followed by 3 mg of methoxyflurane (the dose included in one single-use container for inhalation)—methoxyflurane group (3) patients who received ticagrelor LD followed by 5 mg of morphine administered intravenously—morphine group. Participants in the methoxyflurane arm were advised to inhale the medication for approximately 15 minutes between the baseline and 15-minute study time points.

The obtainment of blood samples was scheduled at nine time points: at baseline and 15, 30, 45, 60, 120, 180, 240, and 360 minutes post-LD of ticagrelor. Each study participant underwent the evaluation of platelet reactivity at each time point of the study with the multiple electrode aggregometry (MEA) in both study sites. Platelet reactivity was expressed as the area under the aggregation curve (AUC), which is affected by the total height of the aggregation curve and its slope. AUC is best suited to express the overall platelet activity. The pharmacokinetic profile was studied by assessing serum concentrations of ticagrelor and its active metabolite. Ticagrelor is metabolized primarily by cytochrome P450 3A enzymes and converted to the active metabolite AR-C124910XX. The inhibition potency of the platelet P2Y12 receptor by the metabolite AR-C124910XX and ticagrelor is the same. 17 18 The serum concentrations of ticagrelor and its active metabolite, AR-C124910XX, were analyzed in the Department of Medicinal Chemistry, Nicolaus Copernicus University, using mass spectrometry and liquid chromatography with a Shimadzu 8030 ESI-Triple Quadrupole mass spectrometer and Shimadzu UPLC Nexera X2 system with predefined limits of quantification of 4.69 ng/mL for both ticagrelor and the metabolite

Coronary angiography was performed in all study participants after completion of the blood sampling schedule to avoid the influence of the contrast medium administration on the PD measurements. Only individuals with an ACS Risk score < 140 points measured with the GRACE calculator were enrolled. Any deterioration of the patient's medical condition leading to an immediate coronary angiography resulted in the termination of the patient's participation in the remaining parts of the trial.

### Inclusion and Exclusion Criteria

Patients aged 18 to 80 years, hospitalized in the study sites due to UA and qualified for coronary angiography, were enrolled. Among the major exclusion criteria, concurrent treatment with any P2Y12 receptor inhibitor, opioids, oral anticoagulants, low-molecular weight heparin, as well as active bleeding, history of intracranial hemorrhage, recent gastrointestinal tract bleeding, and second- or third-degree atrioventricular block need to be highlighted. The complete list of the METHANE-SIRIO 4 study inclusion and exclusion criteria has previously been published. 19

### Study Outcomes

The median platelet reactivity between the study arms within 6 hours postticagrelor LD, assessed with MEA, was defined as the primary outcome of the study. Secondary outcomes included the percentage of patients with high platelet reactivity (HPR; defined as platelet reactivity > 46 U based on the assessment with MEA) at predefined study endpoints (baseline and 15, 30, 45, 60, 120, 180, 240 and 360 minutes postticagrelor LD) median time required to achieve adequate inhibition of platelet function and the exposure to ticagrelor and its active metabolite, expressed as the area under the plasma concentration-time curve for ticagrelor ( *AUC*_TPC_ ) and its active metabolite ( *AUC*_MPC_ ) between the study arms.

### Statistical Analysis

The statistical analysis was performed using the Statistica 13.0 package (TIBCO Software Inc., California, United States). Continuous variables were presented as means with standard deviations and medians with interquartile range. The Shapiro–Wilk test demonstrated nonnormal distribution of the investigated continuous variables. Therefore, medians were used as a central tendency index, and nonparametric tests were used for analysis. Comparisons of continuous variables between groups were performed with the Kruskal–Wallis one-way analysis of variance and multiple comparison tests. Categorical variables were expressed as the number and the percentage and were compared using the *χ* ^2^ test, *χ* ^2^ test with Yates' correction, or Fisher's exact test, depending on the group size. Results were considered significant at *p* < 0.05.

## Results

**Fig. 1:**
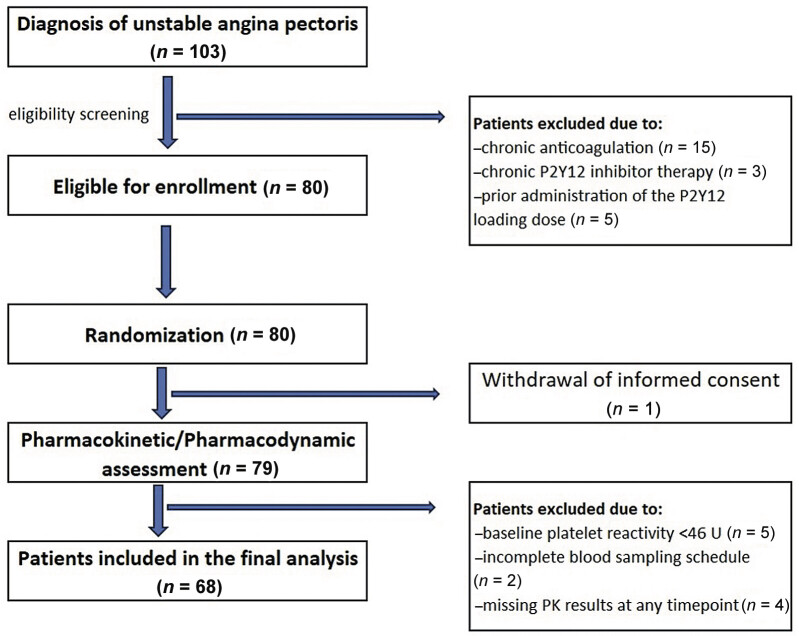
The patients' flowchart throughout the METHANE study.

Overall, 68 participants enrolled in the study, were randomized into the study groups as follows: (1) patients who received 180 mg of ticagrelor ( *n* = 25), (2) patients who received 180 mg of ticagrelor followed by 3 mg of inhaled methoxyflurane ( *n* = 27), and (3) patients who received 180 mg of ticagrelor followed by 5 mg of morphine administered intravenously ( *n* = 16). The patients' flowchart is presented in Fig. 1 . Baseline population characteristics ( Table 1 ) did not show significant differences among the study groups, except for the rate of diabetes mellitus (DM), which was significantly higher in the methoxyflurane group in comparison with the control group (55.56% vs. 20.83%, respectively, *p* = 0.025). The mean patients' weight was also higher in the methoxyflurane group than it was in the control group (90 [88–99] kg vs. 74 [66–90] kg, *p* = 0.017).

**Table 1:** Baseline characteristics of the METHANE-SIRIO 4 study population showing a good balance between the analyzed study arms

	Ticagrelor (group 1; *n* = 25)		Ticagrelor + methoxyflurane (group 2; *n* = 27)		Ticagrelor + morphine (group 3; *n* = 16)		*p* -Value (Kruskal–Wallis)	*p* -Value		
	Mean ± SD	Median (Q1–Q3)	Mean ± SD	Median (Q1–Q3)	Mean ± SD	Median (Q1–Q3)		1 vs. 2	1 vs. 3	2 vs. 3
Age	67.68 ± 8.01	66 (63–75)	64.74 ± 8.80	67 (60–71)	69.06 ± 7.63	71 (64.5–75.5)	0.248	–	–	–
Weight (kg)	78.57 ± 16.57	74 (66–90)	89.40 ± 14.79	90 (88–99)	83.97 ± 11.02	85.5 (75.25–92)	0.048	0.043	1.0	0.639
Height (cm)	165.91 ± 11.73	165 (156–176)	170.59 ± 9.15	172 (164–176)	169.38 ± 7.10	169.5 (164.5–175.5)	0.269	–	–	–
BMI (kg/m ^2^ )	28.23 ± 3.06	28.13 (26.03–30.02)	30.86 ± 5.40	29.75 (28.39–33.62)	29.19 ± 2.81	28.41 (26.86–31.77)	0.131	–	–	–
	***n*** **/** ***N*** **(%)**		***n*** **/** ***N*** **(%)**		***n*** **/** ***N*** **(%)**					
Females	15/25 (62.5)		10/27 (37.04)		7/16 (43.75)		0.137	–	–	–
History of CAD	6/25 (25)		15/27 (55.56)		5/16 (31.25)		0.522	–	–	–
History of MI	3/25 (12.5)		10/27 (37.04)		2/26 (12.5)		0.054	–	–	–
History of PCI	5/25 (20.83)		11/27 (40.74)		2/16 (12.5)		0.084	–	–	–
History of CABG	0 (0.0)		2/27 (7.41)		2/16 (12.5)		0.230	–	–	–
Hyperlipidemia	22/25 (91.67)		23/27 (85.19)		14/16 (87.5)		0.952	–	–	–
Hypertension	21/25 (87.5)		21/27 (77.78)		15/16 (93.75)		0.389	–	–	–
CKD	0 (0.0)		1/27 (3.70)		1/16 (6.25)		0.490	–	–	–
History of stroke/TIA	2/25 (8.33)		2/27 (7.41)		2/16 (12.5)		0.836			
Diabetes mellitus	5/25 (20.83)		15/27 (55.56)		7/16 (43.75)		0.030	0.025	0.166	0.665
Insulin therapy	0 (0.0)		3/27 (11.11)		2/16 (12.5)		0.205	–	–	–
Active smoker	1/25 (4.17)		6/27 (22.22)		1/16 (6.25)		0.092	–	–	–
History of smoking	11/25 (45.83)		14/27 (51.85)		1,016 (62.5)		0.512	–	–	–
COPD	1/25 (4.17)		2/27 (7.41)		1/16 (6.25)		0.871	–	–	–

Abbreviations: BMI, body mass index; CABG, coronary artery bypass grafting; CAD, coronary artery disease; CKD, chronic kidney disease; COPD, chronic obstructive pulmonary disease; MI, myocardial infarction; PCI, percutaneous coronary intervention; TIA, transient ischemic attack.

Note: Significant differences were found in terms of the rate of DM and body weight.

**Fig. 2:**
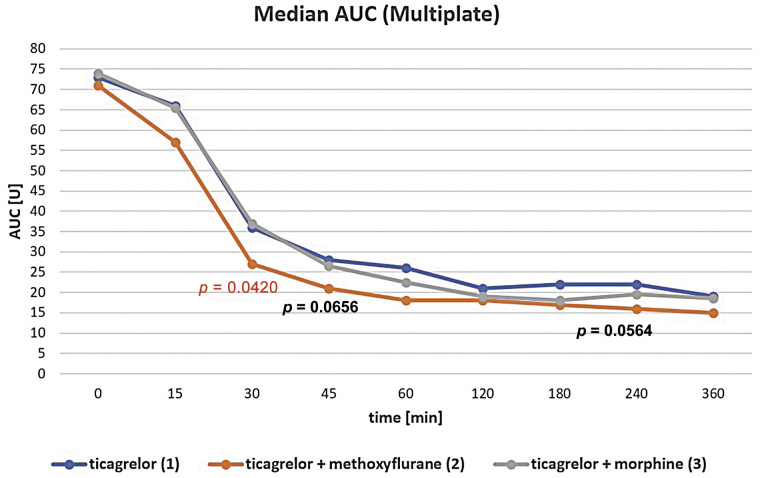
Median platelet reactivity between the study arms in the METHANE-SIRIO 4 study. The *p* -value for significant differences between groups 1 and 2 was marked in red. A trend toward a more favorable PD profile was marked in the bolt.

Median platelet reactivity assessed with MEA and presented as the AUC differed significantly between the control group and the methoxyflurane group at 30-minute postticagrelor LD, and a trend toward lower reactivity was observed at 45 and 240-minute time points ( Table 2 ; Fig. 2 ), which indicates that methoxyflurane has an inhibitory effect on platelet reactivity. No significant differences were observed either between the control group and the morphine group or between the methoxyflurane and the morphine group. Median time to reach low platelet reactivity, defined as AUC below 46 U, and corresponding with the efficacy of the administered ticagrelor, was comparable between the study arms.

**Table 2:** Comparison of pharmacodynamic parameters in the METHANE-SIRIO 4 study

	Ticagrelor (group 1)		Ticagrelor + methoxyflurane (group 2)		Ticagrelor + morphine (group 3)		*p* -Value (Kruskal–Wallis)	*p* -Value		
	Mean ± SD	Median (Q1–Q3)	Mean ± SD	Median (Q1–Q3)	Mean ± SD	Median (Q1–Q3)		1 vs. 2	1 vs. 3	2 vs. 3
AUC 0	76.36 ± 21.82	73 (54–95)	76.56 ± 25.02	71 (57–94)	77.69 ± 21.06	74 (63–100.5)	0.929	–	–	–
AUC 15	66.08 ± 26.54	66 (48–87)	65.04 ± 25.36	57 (45–82)	70.25 ± 23.66	65.5 (58–86)	0.679	–	–	–
AUC 30	47.76 ± 27.79	36 (27–66)	35.59 ± 29.23	27 (20–34)	43.63 ± 26.81	37 (28–53.5)	0.033	0.042	1.0	0.201
AUC 45	34.56 ± 22.95	28 (17–36)	24 ± 27.34	21 (14–24)	38.81 ± 31.91	26.5 (17–50)	0.041	0.066	1.0	0.16
AUC 60	26.64 ± 17.75	26 (16–32)	20.44 ± 19.95	18 (11–22)	30.75 ± 24.56	22.5 (15.5–36.5)	0.084	–	–	–
AUC 120	21.6 ± 13.37	21 (17–25)	18.44 ± 9.51	18 (10–25)	18.13 ± 6.2	19 (14–22.5)	0.596	–	–	–
AUC 180	21.32 ± 12.03	22 (13–28)	17.63 ± 8.71	17 (14–23)	19.44 ± 7.2	18 (14–21.5)	0.471	–	–	–
AUC 240	23.76 ± 16.86	22 (17–26)	15.85 ± 9.77	16 (8–21)	19.13 ± 6.53	19.5 (16.5–22.5)	0.061	–	–	–
AUC 360	19.96 (13.94)	19 (12–25)	17.33 ± 11.07	15 (8–22)	18.75 ± 6.71	18.5 (16–20)	0.71	–	–	–
Time to AUC < 46 U	43.8 ± 46.42	30 (30–45)	31.67 ± 20.01	30 (15–30)	44.06 ± 32.16	30 (30–52.5)	0.338	–	–	–

Abbreviations: AUC, area under the curve; Q1–Q3, interquartile range Q1–Q3; SD, standard deviation.

Note: Platelet reactivity was significantly lower in the ticagrelor group than in the methoxyflurane group 30 minutes postticagrelor LD. Time to achieve adequate platelet inhibition did not differ between the study arms.

**Fig. 3:**
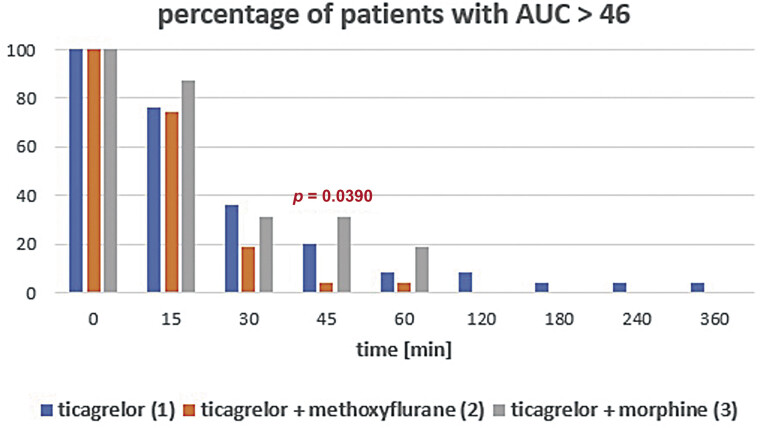
The percentage of patients with high platelet reactivity between study arms in the METHANE-SIRIO 4 study. The *p* -value for a significant difference between groups 2 and 3 was marked in red.

The percentage of HPR patients at predefined study time points revealed a significant difference between the methoxyflurane group versus the morphine group only at 45 minutes following the ticagrelor LD (3.70% vs. 31.25%, respectively, *p* = 0.039; Fig. 3 ). Interestingly, one patient in the ticagrelor group did not achieve adequate platelet inhibition on ticagrelor. Nevertheless, in this case, the coronary angiography showed no significant lesions; therefore, no PCI was required, and the patient was qualified for optimal medical therapy.

Median ticagrelor plasma concentration at study time points is presented in Table 3 . The most pronounced differences were observed between the methoxyflurane group versus the morphine group (45 and 60 minute postticagrelor LD), whereas the only significant difference between the control group versus the methoxyflurane group was found at 240 minutes. The maximum plasma concentration of ticagrelor throughout the study time period was numerically higher in the control group than in the morphine group, but the difference did not reach statistical significance (680.47 [214.41–800.41] ng/mL vs. 474.15 [354.84–643.23] ng/mL, respectively, *p* = 0.066). Consistently, the median ticagrelor's active metabolite concentration ( Table 3 ) revealed significant differences between the methoxyflurane versus the morphine group at 45 and 60 minutes following the administration of ticagrelor LD. Nevertheless, the results did not differ significantly between the control group versus the methoxyflurane group and the control group versus the morphine group at any study time point.

**Table 3:** Median ticagrelor and its active metabolite plasma concentration at study time points

	Ticagrelor (group 1)			Ticagrelor + methoxyflurane (group 2)			Ticagrelor + morphine (group 3)			*p* -Value (Kruskal–Wallis)	*p* -Value		
	Mean ± SD	Median (Q1–Q3)	CV%	Mean ± SD	Median (Q1–Q3)	CV%	Mean ± SD	Median (Q1–Q3)	CV%		1 vs. 2	1 vs. 3	2 vs. 3
**Ticagrelor plasma concentration (0–360 min; ng/mL)**													
Baseline (min)	0	0	NA	0	0	NA	0	0	NA		–	–	–
15	20 ± 29	13 (0.0–16)	145	30 ± 60	14 (11–26)	200	9 ± 6	11 (0–13)	67	0.115	–	–	–
30	171 ± 237	69 (12–178)	139	155 ± 182	112 (45–181)	117	65 ± 55	51 (36–88)	85	0.224	–	–	–
45	329 ± 353	210 (60–470)	107	366 ± 233	350 (163,506)	64	170 ± 127	139 (88–256)	75	0.034	0.426	0.619	0.03
60	464 ± 379	316 (199–751)	82	499 ± 258	515 (261–677)	52	273 ± 203	232 (199–387)	75	0.040	1.0	0.246	0.035
120	533 ± 243	569 (340–756)	46	531 ± 211	496 (371–666)	40	437 ± 216	416 (321–582)	49	0.381	–	–	–
180	4,654 ± 203	453 (329–643)	44	383 ± 155	370 (270–439)	40	440 ± 173	400 (310–567)	39	0.192	–	–	–
240	404 ± 136	402 (289–501)	34	320 ± 140	268 (229–394)	44	403 ± 151	380 (290–472)	38	0.028	0.045	1.0	0.111
360	291 ± 94	290 (216–346)	32	243 ± 87	227 (197–309	36	305 ± 144	261 (214–336)	47	0.176	–	–	–
Ticagrelor maximum concentration	699 ± 257	680 (514–800)	37	592 ± 222	527 (454–692)	38	511 ± 193	474 (355–643)	38	0.065	–	–	–
**Ticagrelor active metabolite plasma concentration (0–360 min; ng/mL)**													
Baseline (min)	0	0	NA	0	0	NA	0	0	NA		–	–	–
15	4 ± 7	0 (0–7)	175	3 ± 9	0 (0–0)	300	1 ± 4	0 (0–0)	400	0.241	–	–	–
30	37 ± 51	17 (7–30)	138	37 ± 53	21 (17–30	145.5	16 ± 11	18 (7–20)	69	0.197	–	–	–
45	101 ± 139	57 (18–92)	138	102 ± 93	80 (53–112)	91.5	41 ± 29	44 (19–52	71	0.015	0.202	0.699	0.014
60	160 ± 176	97 (43–196)	110	181 ± 129	165 (92–223)	71.2	82 ± 66	67 (35–114)	80	0.020	0.448	0.408	0.016
120	290 ± 183	257 (146–444)	63	33 ± 179	305 (208–390)	53.9	211 ± 139	219 (93–263)	66	0.087	–	–	–
180	294 ± 167	271 (187–375)	57	284 ± 142	246 (182–322)	50.2	235 ± 106	232 (132–306)	45	0.598	–	–	–
240	263 ± 100	232 (199–335)	38	240 ± 122	214 (163–275)	50.6	237 ± 110	254 (143,275)	46	0.469	–	–	–
360	225 ± 84	199 (151–305)	37.5	206 ± 96	180 (153–236)	46.7	198 ± 83	213 (140–234)	42	0.666	–	–	–
Metabolite maximum concentration	369 ± 154	328 (244–458)	41.8	349 ± 166	309 (235–399)	47.7	272 ± 115	266 (188–328)	4	0.147	–	–	–
**Time to reach maximum plasma concentration (** ***C***_max_ **)**													
Ticagrelor (min)	126.25 ± 76.98	120 (60–120)	61	94.28 ± 39.93	90 (60–120)	42.4	168.75 ± 66.52	150 (120–180)	39.4	0.001	0.586	0.057	0.001
Metabolite (min)	172.5 ± 77.75	180 (120–210)	45.1	137.5 ± 57.28	120 (120–150)	41.7	198.75 ± 78.13	180 (120–240)	39.3	0.011	0.211	0.8	0.019
**Total exposure to ticagrelor (** ***AUC***_TPC_ **) and active metabolite (** ***AUC***_MPC_ **)**													
*AUC*_TPC_	2,314.71 ± 829.78	2,066.12 (1,785.86–2,900.28)	35.8	2,086.32 ± 760.67	1,991.89 (1,554.67–2,266.95)	36.5	2,018.93 ± 771.13	1,880.88 (1,488.24–2,538.18)	38.2	0.496	–	–	–
*AUC*_MPC_	1,338.74 ± 633.35	1,108.95 (848.39–1,802.02)	47.3	1,329.9 ± 644.86	1,139.75 (934.47–1,425.48)	48.5	1,064.48 ± 501.1	1,125.16 (587.98–1,244.91)	47.1	0.523	–	–	–

Abbreviations: *AUC*_MPC_ , area under the metabolite plasma concentration curve; *AUC*_TPC_ , area under the ticagrelor plasma concentration curve; *C*_max_ , maximum plasma concentration; CV%, coefficient of variation; Q1–Q3, interquartile range Q1–Q3; SD, standard deviation.

Note: Significant differences were observed at 30 and 45 minutes postticagrelor LD between the methoxyflurane and morphine groups for ticagrelor and for the active metabolite. Ticagrelor alone and methoxyflurane groups differed significantly only 4 hours postticagrelor LD in terms of ticagrelor plasma concentration. Time to reach ticagrelor and its active metabolite maximum concentration was significantly longer in the morphine arm than in the methoxyflurane arm. Total exposure to ticagrelor (AUCTPC) and metabolite (AUCMPC) was comparable between the study arms.

Time to reach maximum plasma concentration of ticagrelor and its active metabolite was significantly shorter in the methoxyflurane group than in the morphine group (90 [60–120] minutes vs. 150 [120–180] minutes, *p* = 0.001 and 120 [120–150] minutes vs. 180 [120–240] minutes, *p* = 0.019 for ticagrelor and its active metabolite, respectively). Taking into account the comparison between the control group versus the morphine group, the difference for ticagrelor was not significant (120 [60–120] minutes vs. 150 [120–180] minutes, *p* = 0.059). The area under the plasma concentration curve for ticagrelor ( *AUC*_TPC_ ) and its active metabolite ( *AUC*_MPC_ ), corresponding with the total exposure to ticagrelor and AR-C124910XX, revealed no significant differences between the study arms throughout the analyzed period.

## Discussion

To date, no randomized clinical trials aimed at the direct comparison of the influence of morphine and methoxyflurane on the PK/PD profile in ACS patients have been conducted. Moreover, the aforementioned ongoing ANEMON-SIRIO 3 study is the first trial to evaluate the clinical efficacy of methoxyflurane in an acute setting of ischemic heart disease. As should be expected from an effective analgesic, methoxyflurane exerts some effects on the cardiovascular system, including a decrease in systolic blood pressure and a reduction in the heart rate. 20 Notably, these effects may be considered particularly beneficial in the case of an ACS due to the expected reduction of the workload of the heart associated with the reduced myocardial oxygen demand. 21 Another study, by Høiseth et al, showed no significant impact of methoxyflurane on hemodynamic parameters such as stroke volume, cardiac output, or mean arterial pressure when compared with healthy volunteers who received fentanyl. 22 With its µ-receptor unrelated mechanism of action, methoxyflurane is free of gastrointestinal adverse events typically present in individuals treated with morphine, such as nausea or vomiting, directly influencing the absorption and the latter serum concentration of oral medications. 16 The safety of methoxyflurane administration was evaluated in a study by Jacobs conducted in Australia. Among a total of 17,629 patients who received at least one dose of methoxyflurane, no increase in the rates of ischemic heart disease, renal dysfunction, diabetes, or cancer was observed in comparison with the control group. 23

The impact of inhalational anesthetics on platelet function was evaluated in a series of historical laboratory and clinical trials. A study by Ueda showed a significant reduction of ADP-induced canine platelet aggregation after the administration of commonly used concentrations of several agents, including nitrous oxide, halothane, diethyl ether, or methoxyflurane. 24 O'Brien et al found that the platelet aggregation during major thoracic surgical procedures decreased if inhaled anesthetics such as halothane were used. 25 Similar outcomes were observed in studies by Sweeney and Williams, and Rosen et al, with the most pronounced effects of halothane. 26 27 Volatile anesthetics were also found to increase the bleeding time in patients undergoing surgical operations in studies by Kokores et al, Dalsgaard-Nielsen et al, and Fyman et al. 28 29 30 All these studies provide strong evidence for the potential use of inhalation anesthetics, including methoxyflurane, for pain control in ACS patients.

The proposed study showed significant deterioration of the PK/PD profile of ticagrelor and its active metabolite in ACS patients treated with morphine in comparison with methoxyflurane or no analgesia. Herein, consistently with PD studies of the influence of inhaled anesthetics on platelet reactivity, a beneficial additive antiaggregatory effect of methoxyflurane in patients treated with ticagrelor could be observed. Compared with morphine, methoxyflurane increases ticagrelor's and its active metabolite concentration and shortens the time to maximum plasma concentration, potentially allowing for a quicker therapeutic effect. Contrary to the morphine effect in ACS patients who received ticagrelor, 3 in the METHANE-SIRIO 4 trial, no significant differences were found in terms of platelet reactivity between patients who received ticagrelor and morphine versus ticagrelor alone. This phenomenon has been previously described by Hobl et al 31 in healthy volunteers, in whom morphine administration had no effect on the efficacy of ticagrelor to block the P2Y12 receptor. It has been postulated that in individuals with initially low platelet reactivity, lower concentrations of ticagrelor and its active metabolite are sufficient to effectively reduce platelet reactivity. 10 This finding should be related to the baseline characteristics of the METHANE-SIRIO 4 study population, as we included only patients diagnosed with UA, not with an acute myocardial infarction, which resulted in the lower baseline platelet reactivity. In the proposed study, the rate of DM in the methoxyflurane group was significantly higher than in the control group, which may have influenced the observed results. Patients with DM have been found to be characterized by increased systemic inflammatory processes, leading to the hyperactivation of platelets. Moreover, in DM patients, a weaker response to antiplatelet agents such as ticagrelor can be expected. In the face of that, more pronounced differences between the study arms might have been possible in a better-balanced population. 32 33 34

### Study Limitations

The METHANE-SIRIO 4 study included only patients with the diagnosis of UA, which is associated with significantly lower baseline platelet reactivity than that observed in the case of an acute MI. Moreover, owing to the relatively low number of participants enrolled in the trial, which may have influenced the outcomes, statistical significance was observed only in particular parameters. It needs to be underlined that due to the self-administration of methoxyflurane, the exact amount of the medication cannot be defined, and its use may not always guarantee optimal patient cooperation. Additionally, due to the different forms of the administered medications (inhaled methoxyflurane and intravenous morphine), no blinding of the treatment was possible.

## Conclusion

To the best of our knowledge, the proposed trial is the first one aimed at evaluating the impact of methoxyflurane on the PK and PD of P2Y12 receptor inhibitors in ACS patients. Based on the results of the METHANE-SIRIO 4 study, co-administration of methoxyflurane as an analgesic agent in ACS may not only allow for minimizing the occurrence of negative morphine-related effects, but also has the additional effect of inhibiting platelet reactivity. The PK and PD profile of ticagrelor and its active metabolite is clearly improved in comparison with patients who receive opioids. Further large-scale clinical studies, especially in patients with HPR, would be required to support the aforementioned findings and assess the safety and efficacy of treatment in patients with ACS.

## Notes

**What is known about this topic?**

- Use of morphine has been found to worsen PK and PD of oral P2Y12 inhibitors in acute coronary syndrome patients.
- To date, multiple measures to overcome the morphine effect have been tested, but their clinical utility is limited.

**What does this paper add?**

- Platelet reactivity in acute coronary patients treated with ticagrelor and methoxyflurane was lower than in the ticagrelor alone group.
- Methoxyflurane allows for the improvement of the pharmacokinetic and pharmacodynamic profile of ticagrelor.
